# pH-Responsive i-motif Conjugated Hyaluronic Acid/Polyethylenimine Complexes for Drug Delivery Systems

**DOI:** 10.3390/pharmaceutics11050247

**Published:** 2019-05-27

**Authors:** Gyeong Jin Lee, Tae-il Kim

**Affiliations:** 1 Department of Biosystems & Biomaterials Science and Engineering, College of Agriculture and Life Sciences, Seoul National University, 1 Gwanak-ro, Gwanak-gu, Seoul 08826, Korea; kkk347@snu.ac.kr; 2 Research Institute of Agriculture and Life Sciences, Seoul National University, 1 Gwanak-ro, Gwanak-gu, Seoul 08826, Korea

**Keywords:** hyaluronic acid, i-motif, pH-responsive, polyethylenimine, nanostructure, drug delivery systems

## Abstract

i-motif is cytosine (C)-rich oligonucleotide (ODN) which shows pH-responsive structure change in acidic condition. Therefore, it has been utilized for the trigger of intercalated drug release, responding to environmental pH change. In this study, 2.76 molecules of i-motif binding ODNs (IBOs) were conjugated to each hyaluronic acid (HA) via amide bond linkages. Synthesis of HA-IBO conjugate (HB) was confirmed by FT-IR and agarose gel electrophoresis with Stains-All staining. After hybridization of HB with i-motif ODN (IMO), it was confirmed that doxorubicin (DOX) could be loaded in HB-IMO hybrid structure (HBIM) with 65.6% of drug loading efficiency (DLE) and 25.0% of drug loading content (DLC). At pH 5.5, prompt and significant DOX release from HBIM was observed due to the disruption of HBIM hybrid structure via i-motif formation of IMO, contrary to pH 7.4 condition. Then, HBIM was complexed with low molecular weight polyethylenimine (PEI1.8k), forming positively charged nanostructures (Z-average size: 126.0 ± 0.4 nm, zeta-potential: 16.1 ± 0.3 mV). DOX-loaded HBIM/PEI complexes displayed higher anticancer efficacy than free DOX in A549 cells, showing the potential for pH-responsive anticancer drug delivery systems.

## 1. Introduction

It was reported that specific cytosine (C)-rich oligonucleotide (ODN) can form non-canonical structures called i-motif, which are tetramer structures of cytosine-rich sequences constructed by parallel-stranded duplex of C·CH^+^ (protonated C) pairing in low pH conditions [1,2]. In physiological conditions, i-motif ODN (IMO) can be hybridized with its complementary sequence, i-motif binding ODN (IBO), via normal Watson-Crick base paring [3,4]. Therefore, the heterocyclic anticancer agent, doxorubicin (DOX) can be loaded by intercalation into base planes formed by hybridization of IMO and IBO. Intercalated DOX can be released from IMO-IBO hybrid structure in acidic conditions by disruption of the hybrid structure via i-motif formation of IMO [5].

Several studies using the pH-responsive drug release ability of i-motif system have been reported [6,7,8,9]. An i-motif and G-quadruplex-modified gold nanoparticle (AuNP) was developed for anti-tumor therapy via chemotherapy by DOX, photothermal therapy (PTT) by AuNP, and photodynamic therapy (PDT) by photosensitizer. In acidic conditions, i-motif formation caused AuNP to release DOX by disintegrating the intercalation and to aggregate itself, improving PTT efficiency [8]. In another case, i-motif was grafted on exosome using biotin-streptavidin interaction to deliver DOX by intercalation in their double-stranded structure. In acidic condition, DOX release from exosome was triggered by the formation of i-motif, disintegrating double-stranded DNA structure [9]. 

Hyaluronic acid (HA) is an anionic, non-sulfated polysaccharide with a linear structure composed of D-glucuronic acid and *N*-acetyl-D-glucosamine units. As a family of glycosaminoglycan, HA is a primary component of extracellular matrix and possesses biodegradable, biocompatible, non-toxic, non-immunogenic, and non-inflammatory properties [10,11,12]. HA can be modified chemically by using abundant reactive groups such as carboxyl groups. In addition, it was reported that HA can bind to CD44 receptors overexpressed in many cancer cells such as prostate cancer and breast cancer, showing the targeting ability to these cancer cells [13]. Therefore, HA has been considered and utilized as a versatile natural material in many fields of biomedical applications including drug/gene delivery systems and tissue engineering scaffolds [11,12,14,15]. 

In this work, the pH-responsive hybridization and denaturation properties of i-motif were introduced to HA for drug delivery systems for the first time, to the best of our knowledge. HA is expected to improve the stability of ODN structure and the targeting ability to cancer cells. i-motif binding ODNs (IBOs) were conjugated to HA, synthesizing HA-IBO conjugate (HB). Hybridization of HB and IMO (HBIM formation), DOX loading and pH-responsive release of HBIM structure were examined. To improve the cellular uptake and the stability of HBIM structure, HBIM was complexed with PEI1.8k via electrostatic interaction, forming positively charged nanostructures (HBIM/PEI). Finally, anticancer activity of DOX-loaded HBIM/PEI was investigated. 

## 2. Materials and Methods 

### 2.1. Materials

Sodium hyaluronate (HA, 10.8 kDa) was purchased from Lifecore Biomedical LLC (MN, USA). Oligonucleotides (ODN) (i-motif ODN (IMO, 5′-CCCTAACCCTAAAAAAA-NH_2_-3′) and i-motif binding ODN (IBO, 5′-TTTTTTTAGGGTTAGGG-NH_2_-3′)) were purchased from Bioneer (Daejeon, Korea). Doxorubicin (DOX) was purchased from MedChemExpress LLC (Monmouth Junction, NJ, USA). Polyethylenimine (1800 Da, PEI1.8k), agarose, 2-[4-(2-hydroxyethyl)piperazine-1-yl]ethanesulfonic acid (HEPES), 1-ethyl-3-(3-dimethylaminopropyl)carbodiimide hydrochloride (EDC), ethidium bromide (EtBr), sodium acetate, 3-[4-5-dimethylthiazol-2-yl]2,5-diphenyltetrazolium bromide (MTT), Stains-All, and N-hydroxysuccinimide (NHS) were purchased from Sigma Aldrich (St. Louis, MO, USA). Tris base, dimethyl sulfoxide (DMSO), and hydrochloric acid were purchased from Merck (Darmstadt, Germany). Tris-Ethylenediaminetetraacetic acid (TE) buffer was purchased from Promega (Madison, WI, USA). Dulbecco’s modified Eagle’s medium (DMEM), Dulbecco’s phosphate-buffered saline (DPBS), fetal bovine serum (FBS), penicillin/streptomycin (P/S), and trypsin-EDTA (0.25%) were purchased from Invitrogen (Carlsbad, CA, USA). All other chemicals were purchased and used without further purification. 

### 2.2. Confirmation of ODN Hybridization and Its pH-Responsive Behavior

To confirm the ability of hybridization between IMO and IBO, agarose gel electrophoresis was performed. IMO, IBO, and IMO-IBO hybrid were electrophoresed in 5% agarose gel containing EtBr (5 μg/mL). The electrophoresis was run at 80 V for 20 min (Mupid-2plus, Takara Bio Inc., Shiga, Japan). The ODN and hybrid bands were visualized with UV illuminator (GelDoc XR+ gel documentation system, Bio-Rad, Hercules, CA, USA).

ODN hybridization was further examined by EtBr binding assay. The ODNs and IBO-IMO hybrid (TE buffer) were incubated at different temperatures (25 °C and 37 °C) or in different pH conditions (pH 5.5 and pH 7.4) for 30 min, respectively. Then, 100 μL of each sample was transferred to a black plate and treated with 5 μL of EtBr (0.05 μg/μL) for 5 min. The change of EtBr fluorescence was measured (excitation: 526 nm, emission: 605 nm) using a microplate reader (Synergy H1, BioTek, Winooski, VT, USA). All measurements were performed in triplicate. 

### 2.3. Interaction of ODN Hybrid with DOX 

To investigate the interaction of IBO-IMO hybrid (IBM) with DOX, IBM was incubated with DOX for 24 h with continuous shaking. Then, the mixture was transferred to a black plate and the fluorescence intensity of DOX was measured (excitation: 480 nm) in DMSO and deionized water, respectively.

### 2.4. Synthesis of HA-IBO Conjugate (HB)

IBOs were conjugated to HA with EDC/NHS chemistry. Following this, 30 mg of HA was dissolved in 7.5 mL of PBS (pH 7.4) and reacted with IBO (10 molar excess of HA) using EDC/NHS (4 molar excess of carboxyl groups of HA). After 24 h reaction at 4 °C, the reaction solution was centrifuged with Amicon Ultra-15 centrifugal filters (MWCO 100 kDa, Merck, Darmstadt, Germany) at 3000 relative centrifugal force (RCF), 25 °C for 30 min to remove unreacted materials and byproducts. It was washed with nuclease-free water (nfH_2_O) twice. Finally, the product above the filter membrane, HB was collected and lyophilized.

### 2.5. Confirmation of IBO Conjugation to HA

To confirm the conjugation of IBO to HA, Fourier transform infra-red spectrometry (FT-IR, Nicolet 6700, Thermo Scientific, Waltham, MA, USA) analysis was performed in attenuated total reflectance (ATR) mode between 4000–650 cm^−1^. In addition, agarose gel electrophoresis was conducted by using 0.005% Stains-All solution (50% ethanol). Following this, 3% agarose gel with 5 mm thickness was prepared for efficient staining with Stains-All. Before electrophoresis, pre-running was performed to eliminate any impurities in agarose gel for 6 h. The electrophoresis was conducted at 80 V for 20 min. Then, the gel was stained in Stains-All solution for 2 days at room temperature without light. After staining, it was incubated in deionized water overnight and de-stained in the presence of light for 1 h. 

The concentration of IBO in HB was determined by measuring IBO absorbance at 260 nm. Then, the degree of IBO conjugation in HB was calculated from the absorbance results. 

### 2.6. DOX Loading in HB-IMO hybrid (HBIM)

During the process, 10 mg of HB was dissolved in 5 mL TE buffer, and IMO (equivalent amount to IBO amount in HB) was added to HB solution to form HB-IMO hybrid (HBIM). Then, 4.2 mg of DOX was dissolved in nfH_2_O and added to HBIM solution. It was incubated for 24 h with continuous shaking at room temperature in dark condition. After 24 h, HBIM/DOX solution was purified three times by Amicon Ultra-15 centrifugal filters (MWCO 3 kDa, 3000 RCF, 30 min, 25 °C). Remaining solution above the filter membrane was re-suspended with nfH_2_O and lyophilized to obtain DOX-loaded HBIM (HBIM/DOX). 

Drug loading content (DLC) and drug loading efficiency (DLE) of HBIM/DOX were calculated by measuring the fluorescence of DOX loaded on HBIM by using a microplate reader (excitation: 480 nm, emission: 595 nm) after dissolving HBIM/DOX in DMSO.

$$\mathrm{DLC}\;\left(\%\right)=\frac{Weight\;of\;Drug\;Loaded\;on\;HBIM}{Weight\;of\;HBIM/DOX}\times 100$$

$$\mathrm{DLE}\;\left(\%\right)=\frac{Weight\;of\;Drug\;Loaded\;on\;HBIM}{Weight\;of\;Initial\;Feed\;Drug}\times 100$$

### 2.7. pH-Responsive Release of DOX

To investigate pH-responsive DOX release behavior from HBIM, HBIM/DOX was dissolved in HEPES buffer with different pHs (pH 7.4 and 5.5). Then, fluorescence of released DOX was measured every 5 min for 1 h using the microplate reader. During the measurement (six times), the sample plate was shaken continuously. The results were plotted as fluorescence intensity versus time.

### 2.8. Particle Formation of HBIM with PEI1.8k (HBIM/PEI)

To induce a more efficient cellular uptake of HBIM, it was complexed with PEI (1800 Da, PEI1.8k). Then, 1 mg/mL of PEI1.8k solution was mixed with HBIM solution at a weight ratio of 1 (PEI/HBIM) for 30 min at room temperature to make HBIM/PEI complexes. To confirm the formation of complex structure, Z-average size and zeta-potential of the complexes were measured by Zeta-sizer Nano ZS90 (Malvern Instruments, Malvern, UK) with He-Ne laser beam (633 nm) at 25 °C.

### 2.9. Cytotoxicity

The cytotoxicity of the polymers was evaluated by MTT assay. HeLa human cervical adenocarcinoma cells and A549 human lung adenocarcinoma epithelial cells were maintained in DMEM supplemented with 10% FBS and 1% P/S in humidified atmosphere containing 5% CO_2_ at 37 °C. Then, the cells were seeded on 96-well cell culture plates at a density of 1 × 10^4^ cells/well in 100 μL of DMEM (10% FBS and 1% P/S). After achieving 70–80% confluency, the cells were treated with the polymer solutions (serum-free DMEM) for 24 h. Then, the media were exchanged with fresh DMEM (10% FBS and 1% P/S). Then, 25 μL of MTT solution (2 mg/mL in DPBS) were added to each well and further incubation was performed for 2 h. The media was removed carefully and 150 μL of DMSO was added to each well to dissolve formazan crystal formed by proliferating cells. The absorbance was measured at 570 nm by using microplate reader. Results were presented as relative cell viability (RCV, percentage values relative to value of untreated control cells). All experiments were performed in triplicate.

### 2.10. Anticancer Efficacy of HBIM/DOX/PEI

To estimate the anticancer efficacy of HBIM/DOX/PEI, cancer cell-killing activity of HBIM/DOX/PEI was measured by MTT assay in HeLa and A549 cells. The cells were seeded on 96 well cell culture plates at a density of 1 × 10^4^ cells/well in 100 μL of DMEM (10% FBS and 1% P/S). After achieving 70–80% confluency, the cells were treated with the sample solutions (serum-free DMEM) for 24 h. Free DOX was used as a control. Then, the media were exchanged with fresh DMEM (10% FBS and 1% P/S). The further assay procedure was identical to above MTT assay (Section 2.9). 

## 3. Results and Discussion

### 3.1. Hybridization of IMO and IBO

**Figure 1:**
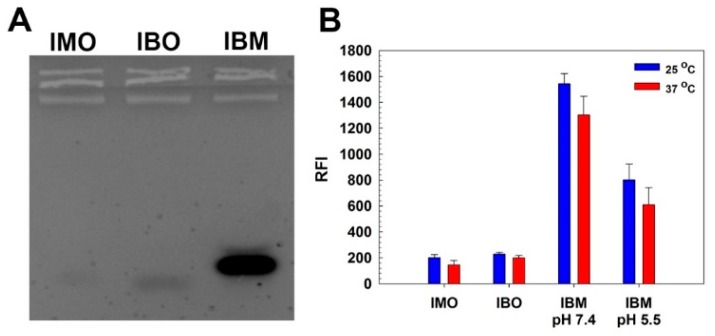
Hybridization behavior of i-motif ODN (IMO) and i-motif binding ODN (IBO). (**A**) Agarose gel electrophoresis result. (**B**) EtBr binding assay result. Relative fluorescence intensity (RFI).

Prior to conjugation of ODNs to HA, hybridization and denaturation behavior of IMO and IBO were investigated by EtBr binding assay. EtBr is known to bind single stranded ODN showing sufficient fluorescence to detect and its fluorescence is significantly increased with binding to double stranded DNA [16]. First, agarose gel electrophoresis was performed to confirm the hybridization of IMO and IBO. In Figure 1A, although IMO and IBO only displayed faint bands on the gel, IBO-IMO hybrid (IBM) showed a strong band, meaning that IMO and IBO can be hybridized via complementary base paring. Less migration of the IBM band than those of IMO and IBO bands also demonstrated the size increase of IBM via hybridization. Hybridization and denaturation of IMO and IBO were further examined by measuring EtBr fluorescence change (Figure 1B). IMO and IBO showed low fluorescence values (~200 relative fluorescence intensity, RFI) due to the binding of EtBr to single stranded ODNs. Abrupt increase of fluorescence from IBM at pH 7.4 was observed, meaning the hybridization of IBO and IMO. At pH 5.5, the fluorescence of IBM was decreased to about 50% value of IBM fluorescence at pH 7.4. This result showed that hybrid structure of IBO and IMO could be denaturated in acidic condition probably due to the formation of i-motif structure of IMO and the following dissociation from IBO. It is also noteworthy that elevated temperature (37 °C) slightly diminished the EtBr fluorescence intensity from hybrids with low melting temperature (44.1 °C provided by the manufacturer) due to the partial denaturation. 

### 3.2. Interaction of IBM with DOX

**Figure 2:**
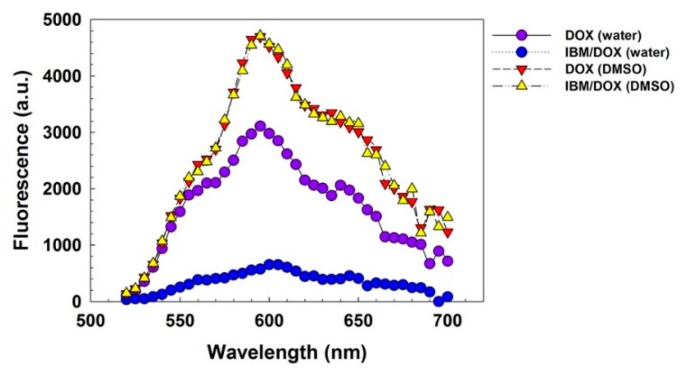
Fluorescence measurement of doxorubicin (DOX) and IBM/DOX in water and dimethyl sulfoxide (DMSO).

It is known that DOX molecules can be intercalated into base planes of hybrid structures, quenching its fluorescence [17]. Therefore, interaction of IBM with DOX was investigated by measuring the DOX fluorescence change. In Figure 2, fluorescence from IBM/DOX in deionized water was much lower than that of free DOX in deionized water (18.5%) and DMSO (12.2%). Fluorescence of DOX is reported to be quenched in water due to the collision of water molecules [18]. On the other hand, when IBM/DOX was dissolved in DMSO, its fluorescence was fully restored to that of free DOX in DMSO, due to the complete release of DOX from denaturated IBM structure in DMSO. This result means that DOX molecules would be efficiently intercalated into base planes of IBM structure in aqueous conditions, showing the high potential for DOX loading. 

### 3.3. Synthesis and Characterization of HA-IBO Conjugate (HB)

In order to improve the stability of IBM structure and to introduce the targeting ability to CD44 receptors in cancer cells [19], HA was utilized for modification of IBO. Conjugation of IBO to HA was conducted through EDC/NHS chemistry. Figure S1 shows the synthesis scheme. To achieve this end, IBO was originally designed to have a primary amine group at 3’-end of the sequence. Carboxyl groups of HA were activated by EDC/NHS chemistry and expected to react with a primary amine group of IBO to form amide bond linkages. After conjugation reaction, unreacted materials and byproducts were removed by ultrafiltration of Amicon filter. Then, the synthesis of HA-IBO conjugate (HB) was confirmed by FT-IR analysis and agarose gel electrophoresis.

**Figure 3:**
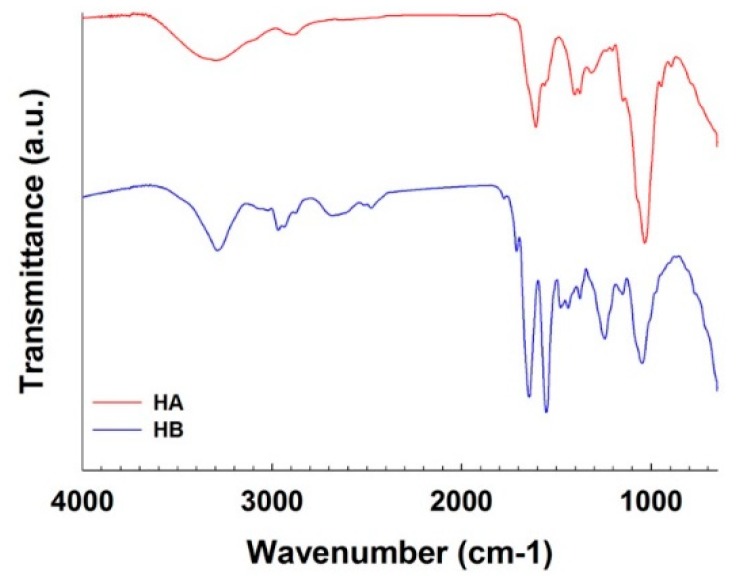
FT-IR spectra of HA and HA-IBO conjugate (HB).

Figure 3 showed FT-IR spectra of HA and HB. In HA spectrum, characteristic IR peaks of HA were observed (C=O stretching: 1561 cm^−1^, C–OH: 1035 cm^−1^, OH: 946 cm^−1^, C–O stretching: 1376 cm^−1^) [20,21]. In HB spectrum, amide 1 peak (1710 cm^−1^) became more distinct, and amide 2 peak (1643 cm^−1^) and C=O stretching peak became more noticeable. On the other hand, the peak assigned to C–OH of carboxyl group was decreased. New peaks from P=O (1244 cm^−1^) and nucleotide base group vibrations (1500–1700 cm^−1^) also appeared [22]. This FT-IR analysis result revealed the formation of amide bond between HA and HB and existence of IBO in HB. 

**Figure 4:**
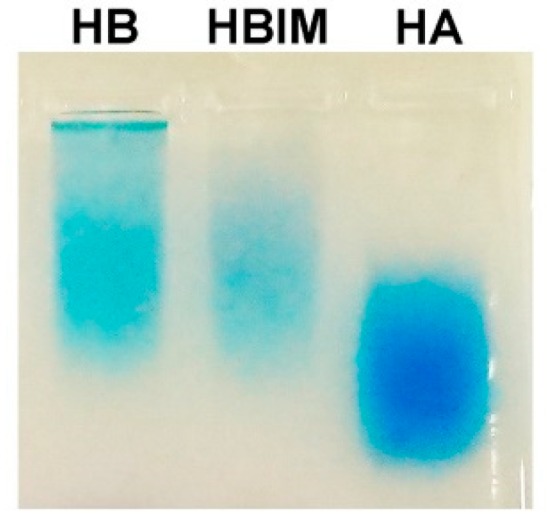
Stains-All staining result of HB, HBIM, and HA in agarose gel electrophoresis.

Agarose gel electrophoresis followed by Stains-All staining was also performed to confirm the conjugation of IBO to HA. Stains-All is known to bind to anionic molecules such as HA and nucleic acids [23,24]. Figure 4 displayed distinct band from HB, hybrid of HB and IMO (HBIM), and HA. HA band was broad, meaning its broad molecular weight distribution. Less migration of HB than HA suggested the increase of molecular size by conjugation of IBO to HA. HBIM band was located at a similar position to HB band even after hybridization of IMO, showing the balance between band retardation by increase of molecular size and band migration by additional negative charges. 

Taken together, considering FT-IR analysis and agarose gel electrophoresis result, it was confirmed that HB was successfully synthesized.

Finally, the degree of IBO conjugation to HA in HB was determined by measuring absorbance of IBO at 260 nm. According to the absorbance result of IBO in HB and comparison with IBO calibration result, it was calculated that average 2.76 IBO molecules were conjugated to one HA chain. 

### 3.4. DOX Loading and Release of HBIM

It was shown that DOX could be loaded in IBM structure via the interaction in Figure 3. In addition, DLE and DLC of HBIM were examined to confirm DOX loading in HBIM structure by measuring DOX fluorescence. DLE and DLC of HBIM/DOX were calculated as 65.6% and 25.0%, respectively, suggesting that DOX also could be efficiently loaded in HBIM structure.

**Figure 5:**
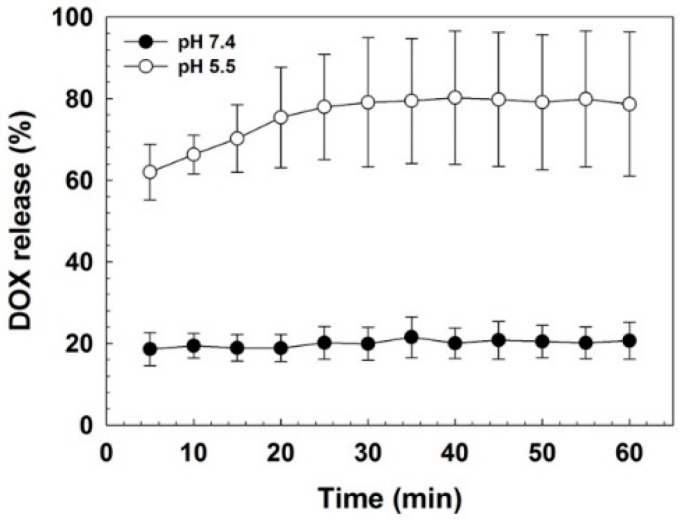
DOX release behavior of HBIM/DOX at pH 7.4 and pH 5.5.

Then, release behavior of DOX in HBIM/DOX was investigated. It was observed by monitoring the change of released DOX fluorescence intensity from HBIM/DOX (Figure 5). At pH 7.4, the amount of released DOX from HBIM/DOX was maintained as about 20% through the measurement period, showing the stability at pH 7.4. However, significant DOX release (about 60%) was observed at pH 5.5 even after 5 min of incubation and it was gradually increased to about 80% (from 30 min incubation). This result means that in acidic conditions, loaded DOX molecules in HBIM structure could be released in pH-responsive way due to the prompt disruption of HBIM hybrid structure by dissociation of IMO via i-motif formation. 

### 3.5. Particle Size and Zeta-Potential of HBIM/PEI

It was found that HBIM could not form particle structure by Zeta-sizer analysis (data not shown) and the HBIM structure would be negatively charged, which may be inefficient for cellular uptake due to electrostatic repulsion, although the DOX loading and release of HBIM structure were already confirmed. Therefore, complex formation was performed to improve the cellular uptake of HBIM via electrostatic interaction with cationic polymer, PEI1.8k. It is also expected that ODNs can be protected from harsh environments (nuclease, pH, salt, etc.) by complex formation with cationic polymers, maintaining DOX loading and delivery efficacy. PEI1.8k was chosen due to its positive charges and low cytotoxicity. HBIM/PEI complex was prepared at a weight ratio of 1 (PEI/HBIM). Z-average particle size and zeta-potential of HBIM/PEI were measured by Zeta-sizer. Z-average particle size was 126.0 ± 0.4 nm and zeta-potential was 16.1 ± 0.3 mV. This result means that HBIM/PEI could form positively charged and nano-sized complex structure, which is considered as prerequisite for efficient interaction with negatively charged cell membrane and facilitated cellular uptake [25]. Figure S2 shows the scheme for the formation of HBIM structure and HBIM/PEI complexes.

### 3.6. Cytotoxicity

**Figure 6:**
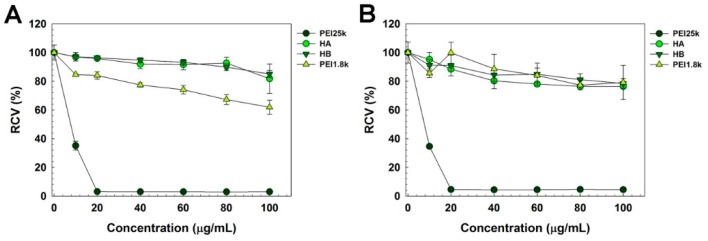
MTT assay result in (**A**) HeLa cells and (**B**) A549 cells (Relative cell viability (RCV)).

Cytotoxicity of polymers was evaluated by MTT assay (Figure 6) in HeLa and A549 cells. In both cells, PEI25k-treated cells showed significant decrease of viability, referring to its high cytotoxicity. However, viability of HA- and HB-treated cells was more than 80% even at high concentration of 100 μg/mL, demonstrating their negligible cytotoxicity. PEI1.8k-treated cells also showed relatively high cell viability. At low concentrations where HBIM/PEI complex was formed (<5 μg/mL), the cell viability was above 90%. 

### 3.7. Anticancer Efficacy of HBIM/DOX/PEI

**Figure 7:**
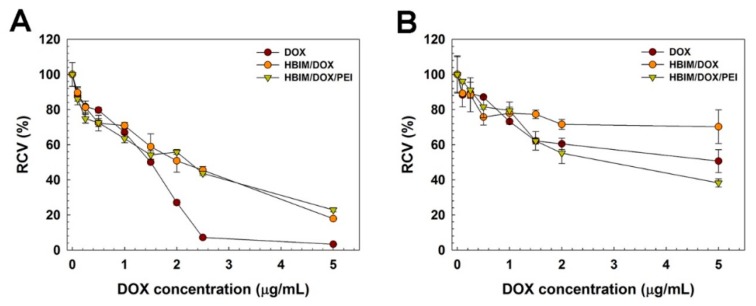
Anticancer activity of HBIM/DOX and HBIM/DOX/PEI in (**A**) HeLa cells and (**B**) A549 cells.

Anticancer efficacy of HBIM/DOX/PEI was examined using MTT assay (Figure 7) in HeLa and A549 cells. In HeLa cells (Figure 7A), Free DOX-treated cell showed rapid decrease of viability and the viability was decreased to about 7% at 2.5 μg/mL, showing its high cytotoxicity to HeLa cells. Both HBIM/DOX and HBIM/DOX/PEI showed similar cell viability decrease dependent on DOX concentration (~20% at 5 μg/mL). In the case of HBIM/DOX, although it could not form nanostructure for efficient cellular uptake, released DOX in acidified culture media would induce similar cytotoxicity. However, in A549 cells (Figure 7B), viability of DOX-treated cells decreased slowly and was still about 50% even at 5 μg/mL, contrary to the HeLa cell result. It was thought that multidrug-resistance (MDR) activity of A549 cells [26,27,28] would export the internalized DOX molecules by transporter proteins such as p-glycoprotein, leading to the reduced anticancer efficacy of DOX. Viability of HBIM/DOX-treated cells was maintained as about 70% even at 5 μg/mL. Interestingly, HBIM/DOX/PEI-treated cells showed higher decrease of viability than free DOX-treated cells (38.2% at 5 μg/mL). These results suggest that HBIM/DOX/PEI would deliver DOX into cancer cells efficiently, partially overcoming their MDR activity by forming nanostructure [29,30]. 

### 3.8. Cellular Uptake of HBIM and HBIM/PEI Complexes in HeLa cells

In order to identify the targeting ability of HBIM to CD44 expressing HeLa cells [31], cellular uptake efficiency of HBIM and HBIM/PEI complexes was examined by flow cytometry. Flow cytometry analysis was performed by measuring the cellular uptake efficiency of HBIM/DOX and HBIM/DOX/PEI complexes without or with free HA (HA competitive inhibition assay [32]). As shown in Figure S3, cellular uptake efficiency of HBIM/DOX and HBIM/DOX/PEI complexes was greatly reduced in HA-pretreated cells (29.8% → 11.8%, 60.1% → 37.0%, respectively) in comparison with normal cell results, meaning the HA receptor (CD44) mediated endocytosis of HBIM and HBIM/PEI complexes. Interestingly, HBIM/DOX/PEI complexes showed much higher cellular uptake efficiency (60.1%) than HBIM/DOX (29.8%), which suggested that the positively charged and nano-sized complexes could be more efficiently internalized into cells, as expected.

## 4. Conclusions

In this work, pH-responsive hybridization and denaturation behavior of i-motif were introduced to HA for drug delivery systems. i-motif binding ODNs were conjugated to HA, synthesizing HB. HB and IMO could form hybrid structure (HBIM), enabling the loading of DOX via intercalation into base planes. HBIM showed pH-responsive DOX release behavior. To improve the cellular uptake and the stability of HBIM structure, HBIM was complexed with PEI1.8k via electrostatic interaction, forming positively charged nanostructures. It was found that HBIM/DOX/PEI showed higher anticancer activity than free DOX in A549 cells, suggesting the possibility of overcoming MDR activity of cancer cells. These results show the potential of HBIM/PEI nanostructure for pH-responsive anticancer drug delivery systems. 

## Supplementary Materials

The following are available online at https://www.mdpi.com/1999-4923/11/5/247/s1, Figure S1: Scheme for the synthesis of HA-IBO conjugate (HB) with EDC/NHS chemistry. Figure S2: Scheme for the formation of HBIM structure via hybridization of HB and IMO, and the formation of HBIM/PEI complexes via electrostatic interaction of negatively charged HBIM and positively charged PEI1.8k. Figure S3: Cellular uptake efficiency of HBIM/DOX and HBIM/DOX/PEI complexes in HeLa cells.

## References

[1] Leroy J.L., Guéron M., Mergny J.L., Hélène C. (1994). Intramolecular folding of a fragment of the cytosine-rich strand of telomeric DNA into an I-motif. Nucleic Acids Res..

[2] Guéron M., Leroy J.L. (2000). The i-motif in nucleic acids. Curr. Opin. Struct. Biol..

[3] Phan A.T., Mergny J.-L. (2002). Human telomeric DNA: G-quadruplex, i-motif and Watson-Crick double helix. Nucleic Acids Res..

[4] Benabou S., Aviñó A., Eritja R., González C., Gargallo R. (2014). Fundamental aspects of the nucleic acid i-motif structures. RSC Adv..

[5] Xu C., Zhao C., Ren J., Qu X. (2011). PH-controlled reversible drug binding and release using a cytosine-rich hairpin DNA. Chem. Commun..

[6] Song L., Ho V.H.B., Chen C., Yang Z., Liu D., Chen R., Zhou D. (2013). Efficient, pH-Triggered Drug Delivery Using a pH-Responsive DNA-Conjugated Gold Nanoparticle. Adv. Healthc. Mater..

[7] Kim J., Lee Y.M., Kang Y., Kim W.J. (2014). Tumor-Homing, Size-Tunable Clustered Nanoparticles for Anticancer Therapeutics. ACS Nano.

[8] Park H., Kim J., Jung S., Kim W.J. (2018). DNA-Au Nanomachine Equipped with i-Motif and G-Quadruplex for Triple Combinatorial Anti-Tumor Therapy. Adv. Funct. Mater..

[9] Kim J.Y., Song J., Jung H., Mok H. (2018). I-motif-coated exosomes as a pH-sensitive carrier for anticancer drugs. Appl. Biol. Chem..

[10] Mero A., Campisi M., Mero A., Campisi M. (2014). Hyaluronic Acid Bioconjugates for the Delivery of Bioactive Molecules. Polymers.

[11] Khaing Z.Z., Seidlits S.K. (2015). Hyaluronic acid and neural stem cells: implications for biomaterial design. J. Mater. Chem. B.

[12] Kim J.H., Moon M.J., Kim D.Y., Heo S.H., Jeong Y.Y. (2018). Hyaluronic acid-based nanomaterials for cancer therapy. Polymers (Basel).

[13] Saravanakumar G., Deepagan V.G., Jayakumar R., Park J.H. (2014). Hyaluronic acid-based conjugates for tumor-targeted drug delivery and imaging. J. Biomed. Nanotechnol..

[14] Khunmanee S., Jeong Y., Park H. (2016). Crosslinking method of hyaluronic-based hydrogel for biomedical applications. J. Tissue Eng..

[15] Tiwari S., Bahadur P. (2019). Modified hyaluronic acid based materials for biomedical applications. Int. J. Biol. Macromol..

[16] Olmsted J., Kearns D.R. (1977). Mechanism of ethidium bromide fluorescence enhancement on binding to nucleic acids. Biochemistry.

[17] Mohan P., Rapoport N. (2010). Doxorubicin as a Molecular Nanotheranostic Agent: Effect of Doxorubicin Encapsulation in Micelles or Nanoemulsions on the Ultrasound-Mediated Intracellular Delivery and Nuclear Trafficking. Mol. Pharm..

[18] Rapoport N., Pitina L. (1998). Intracellular Distribution and Intracellular Dynamics of a Spin-Labeled Analogue of Doxorubicin by Fluorescence and EPR Spectroscopy. J. Pharm. Sci..

[19] Zhong Y., Goltsche K., Cheng L., Xie F., Meng F., Deng C., Zhong Z., Haag R. (2016). Hyaluronic acid-shelled acid-activatable paclitaxel prodrug micelles effectively target and treat CD44-overexpressing human breast tumor xenografts in vivo. Biomaterials.

[20] Donghui F., Beibei W., Zheng X., Qisheng G. (2006). Determination of hyaluronan by spectroscopic methods. J. Wuhan Univ. Technol. Sci. Ed..

[21] Wu Y. (2012). Preparation of low-molecular-weight hyaluronic acid by ozone treatment. Carbohydr. Polym..

[22] Pershina A.G., Ogorodova L.M., Magaeva A.A., Itin V.I., Naiden E.P., Izaak T.I., Shchegoleva N.N., Sazonov A.E. (2015). Sequence-selective binding of oligonucleotides to superparamagnetic cobalt ferrite nanoparticles: a new way to fabricate functional nanoconjugates. RSC Adv..

[23] Volpi N., Maccari F. (2002). Detection of submicrogram quantities of glycosaminoglycans on agarose gels by sequential staining with toluidine blue and Stains-All. Electrophoresis.

[24] Cong W.-T., Ye W.-J., Chen M., Zhao T., Zhu Z.-X., Niu C., Ruan D., Ni M.-W., Zhou X., Jin L.-T. (2013). Improved staining of phosphoproteins with high sensitivity in polyacrylamide gels using Stains-All. Electrophoresis.

[25] Fröhlich E. (2012). The role of surface charge in cellular uptake and cytotoxicity of medical nanoparticles. Int. J. Nanomed..

[26] Lehmann T., Köhler C., Weidauer E., Taege C., Foth H. (2001). Expression of MRP1 and related transporters in human lung cells in culture. Toxicology.

[27] Salomon J.J., Ehrhardt C. (2011). Nanoparticles attenuate P-glycoprotein/MDR1 function in A549 human alveolar epithelial cells. Eur. J. Pharm. Biopharm..

[28] Kim T., Park J., Kim T.-i. (2019). Cholic Acid-Conjugated Methylcellulose-Polyethylenimine Nano-Aggregates for Drug Delivery Systems. Nanomaterials.

[29] Huang Y., Cole S.P.C., Cai T., Cai Y. (2016). Applications of nanoparticle drug delivery systems for the reversal of multidrug resistance in cancer. Oncol. Lett..

[30] Yuan Y., Cai T., Xia X., Zhang R., Chiba P., Cai Y. (2016). Nanoparticle delivery of anticancer drugs overcomes multidrug resistance in breast cancer. Drug Deliv..

[31] Wang K., Zeng J., Luo L., Yang J., Chen J., Li B., Shen K. (2013). Identification of a cancer stem cell-like side population in the HeLa human cervical carcinoma cell line. Oncol. Lett..

[32] Lee H., Mok H., Lee S., Oh Y.-K., Park T.G. (2007). Target-specific intracellular delivery of siRNA using degradable hyaluronic acid nanogels. J. Control. Release.

