# Supplementary material for: pH-Responsive i-motif Conjugated Hyaluronic Acid/Polyethylenimine Complexes for Drug Delivery Systems
Source: Pharmaceutics. 2019 May 27;11(5):247. doi: 10.3390/pharmaceutics11050247 (PMC6572266; doi:10.3390/pharmaceutics11050247)
Supplement: Supplementary file 1 [file pharmaceutics-11-00247-s001.pdf]

pharmaceutics

MDPI

Supplementary Materials: pH-Responsive i-motif Conjugated Hyaluronic Acid/Polyethylenimine Complexes for Drug Delivery Systems

Gyeong Jin Lee and Tae-il Kim

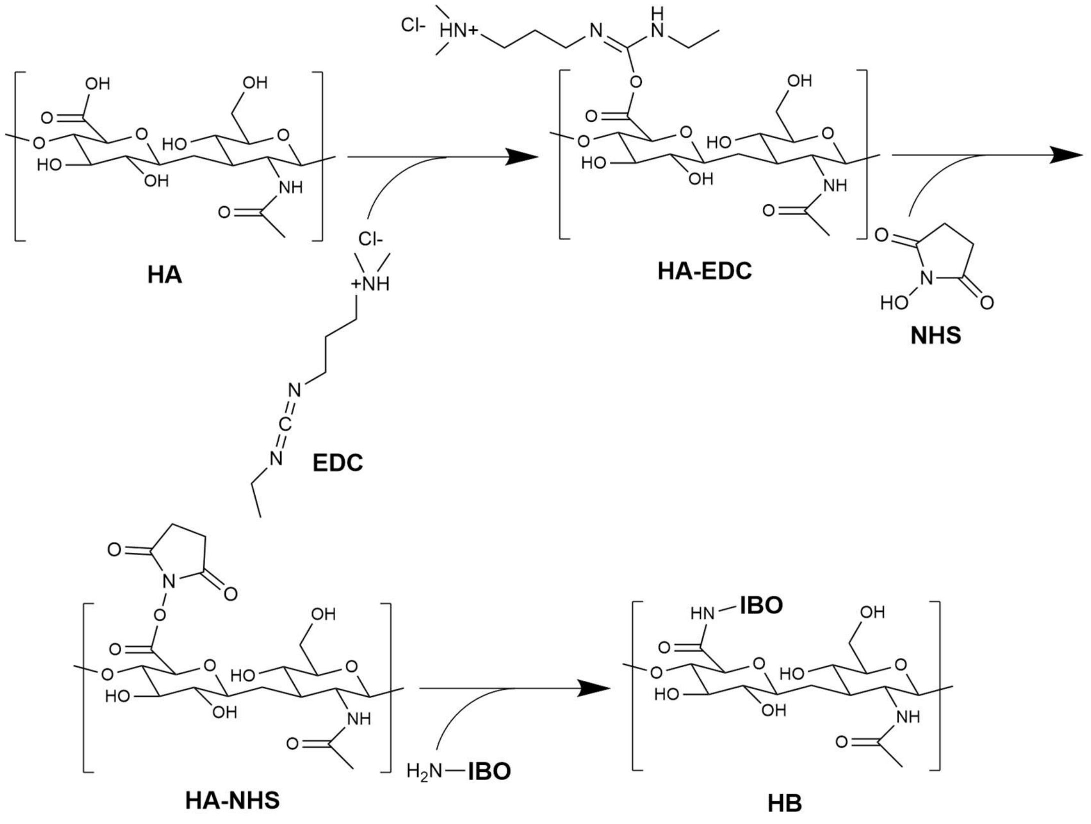

Figure S1. Scheme for the synthesis of HA-IBO conjugate (HB) with EDC/NHS chemistry. Carboxyl acid groups of HA are activated by EDC via forming HA-EDC (O-acyl urea intermediate). O-acyl urea of HA-EDC is replaced with NHS via nucleophilic attack of hydroxyl group of NHS to activated carboxyl groups of HA, forming HA-NHS ester. Finally, primary amine of IBO reacts with HA-NHS, conjugating IBO to HA (synthesis of HB) via amide bond linkages.

pharmaceutics

MDPI

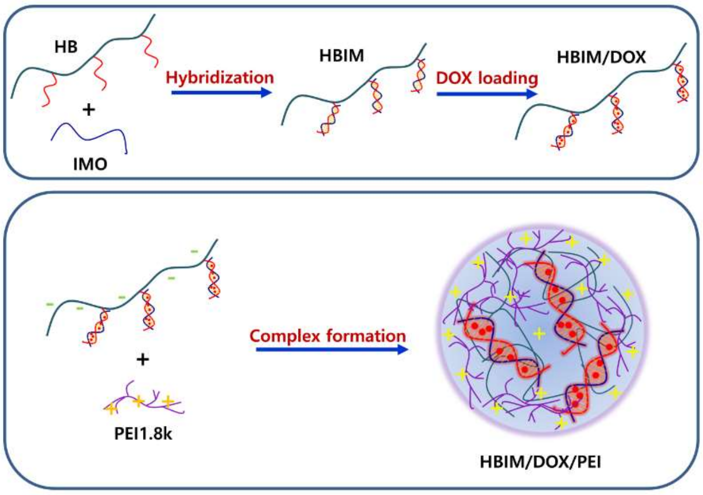

Figure S2. Scheme for the formation of HBIM structure via hybridization of HB and IMO, and the formation of HBIM/PEI complexes via electrostatic interaction of negatively charged HBIM and positively charged PEI1.8k. DOX molecules (red dots) can be intercalated into hybridized base planes of HBIM.

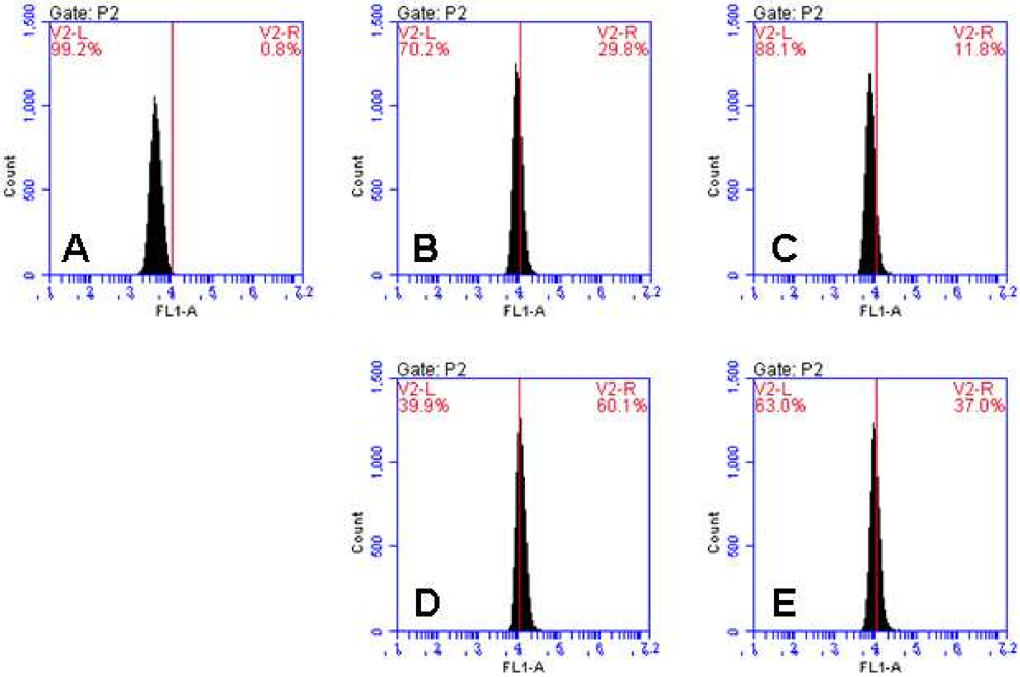

Figure S3. Cellular uptake efficiency of HBIM/DOX and HBIM/DOX/PEI complexes in HeLa cells. Cell only (**A**), HBIM/DOX (**B**), HBIM/DOX in HA condition (**C**), HBIM/DOX/PEI (**D**), and HBIM/DOX/PEI in HA condition (**E**). Cells were seeded at a density of 3×10^5 cells/well in a 6-well plate and grown to reach 70–80% confluency. Prior to sample treatment, cells were pretreated with DMEM or DMEM containing HA solution (20 mg/mL) for 1 h. Then, HBIM/DOX or HBIM/DOX/PEI complexes (DOX = 2 µg/mL) were treated to the cells for 3 h. After removal of media, the cells were washed with ice-cold DPBS twice and trypsinized. The detached cells were suspended in 2 mL DPBS and re-suspended after centrifuge. The cellular uptake was determined by measuring the fluorescence of loaded DOX by BD Accuri C6 flow cytometer (Becton Dickinson, Piscataway, NJ, USA) at a minimum of 1×10^4 cells gated per sample. Analysis was performed by BD Accuri C6 software (Becton Dickinson, Piscataway, NJ, USA).
